# Measurement of soil bacterial colony temperatures and isolation of a high heat-producing bacterium

**DOI:** 10.1186/1471-2180-13-56

**Published:** 2013-03-11

**Authors:** Kenji Tabata, Fuminori Hida, Tomoyuki Kiriyama, Noriaki Ishizaki, Toshiaki Kamachi, Ichiro Okura

**Affiliations:** 1 Frontier Research Center, Tokyo Institute of Technology, 4259 Nagatsuta, Midori-ku, Yokohama 226-8501, Japan; 2 Department of Bioengineering, Tokyo Institute of Technology, 4259 Nagatsuta, Midori-ku, Yokohama 226-8501, Japan

**Keywords:** Heat production, Thermogenesis, Thermograph, Growth-independent reaction, Energy-spilling reaction

## Abstract

**Background:**

The cellular temperatures of microorganisms are considered to be the same as those of their surroundings because the cellular volume is too small to maintain a cellular temperature that is different from the ambient temperature. However, by forming a colony or a biofilm, microorganisms may be able to maintain a cellular temperature that is different from the ambient temperature. In this study, we measured the temperatures of bacterial colonies isolated from soils using an infrared imager and investigated the thermogenesis by a bacterium that increases its colony temperature.

**Results:**

The temperatures of some colonies were higher or lower than that of the surrounding medium. A bacterial isolate with the highest colony temperature was identified as *Pseudomonas putida*. This bacterial isolate had an increased colony temperature when it grew at a temperature suboptimal for its growth. Measurements of heat production using a microcalorimeter showed that the temperature of this extraordinary, microcalorimetrically determined thermogenesis corresponded with the thermographically observed increase in bacterial colony temperature. When investigating the effects of the energy source on this thermal behavior, we found that heat production by this bacterium increased without additional biomass production at a temperature suboptimal for its growth.

**Conclusions:**

We found that heat production by bacteria affected the bacterial colony temperature and that a bacterium identified as *Pseudomonas putida* could maintain a cellular temperature different from the ambient temperature, particularly at a sub-optimal growth temperature. The bacterial isolate *P. putida* KT1401 increased its colony temperature by an energy-spilling reaction when the incubation temperature limited its growth.

## Background

Any reaction in a living system is followed by heat production. Monitoring heat production is valuable for investigating metabolic reactions in living systems, and heat production by microorganisms has been extensively investigated [1-5]. Heat production by bacteria is related to their growth phases because the heat produced by bacteria is tightly coupled to their metabolic reactions [1]. Thus, heat output monitoring has been used to determine bacterial growth rates. The heat output of bacteria is characteristic of the particular strain because the amount of heat produced by bacteria is affected by nutrients and the bacterial products and metabolic pathways. In previous studies, heat output measurements were used to characterize bacteria [2,5]. Heat output measurements were also used to investigate the effects of a particular compound in a medium on bacterial growth [6-8].

Detailed studies on the relationships between substrate consumption and biomass production by bacteria have suggested that some bacteria can consume higher amounts of energy without concomitant biomass production [9-12]. In these growth independent reactions, energy sources were converted to heat. Russell called these growth independent reactions energy-spilling reactions [10]. Some bacteria use futile cycles to spill energy. The energy-spilling reaction of *Streptococcus bovis* is mediated by a futile cycle of protons through its cell membrane. A futile cycle between pyruvate and phosphoenolpyruvate was proposed in the metabolic pathway of *Escherichia coli*[13] and another futile cycle between fructose-6-phosphate and fructose-1,6-bisphosphate was proposed in the metabolic pathway of *Streptococcus cremoris*[14]. In the case of an energy-spilling reaction that increases under nitrogen-limited and excess glucose conditions, the energy-spilling reaction is used to reduce glucose toxicity [11]. However, the roles of energy-spilling reactions in many bacteria are not completely understood.

In the case of homeotherms, some growth independent reactions are utilized to maintain a constant body temperature. UCP1, which is located in the mitochondrial inner membrane of brown adipocytes, disrupts the mitochondrial membrane potential without the production of ATP [15]. This UCP1-mediated reaction is considered to play a major role in the thermogenesis of brown adipocytes. However, the effects of the growth independent reactions of bacteria on cellular temperature have not been investigated.

The cellular temperatures of microorganisms have been considered to be the same as those of their surroundings because the cellular volume is too small to maintain a cellular temperature different from the ambient temperature. However, by forming a colony or a biofilm, microorganisms may be able to maintain a cellular temperature that is different from the ambient temperature. Therefore, in this study, we investigated the temperature of bacterial colonies and attempted to isolate bacteria that could increase their colony temperatures above the ambient temperature. For this study, we investigated the colony temperatures of bacteria isolated from soil because the environment of bacteria living in soil is more adiabatic than the environments of bacteria that live in water or intestines.

## Methods

### Bacterial strains and materials

*Pseudomonas putida* TK1401 was isolated from soil and deposited in the International Patent Organism Depository (Agency of Industrial Science and Technology, Japan) under accession no. FERM P-20861. *Pseudomonas putida* KT2440 (ATCC 47054) was obtained from the Global Bioresource Center (ATCC, Manassas, VA, USA). All chemicals were purchased from Wako Pure Chemical Industries, Ltd (Japan).

### Bacterial isolation

Bacteria were isolated from soil samples from the forest and gardens in Kanagawa Prefecture, Japan, during June and October. Most soil samples were slightly moist and brown in color. A soil sample was suspended in 1 ml of distilled water. This suspension was diluted 1:1000 with distilled water and 10 ml of this diluted suspension was inoculated onto a Luria–Bertani (LB) agar plate. The LB agar plate was incubated at 30°C until some colonies had formed. Bacteria that formed colonies were isolated. After single-colony isolation, these bacteria were stored at −80°C.

### Bacterial identification

Total DNA isolation and amplification of the 16S rRNA gene was performed as described by Hiraishi et al. [16]. After purifying the PCR product using a QIAquick PCR Purification kit (QIAGEN GmbH), the nucleotide sequence was determined by a dideoxynucleotide chain-termination method using a Genetic Analyzer 310 (Applied Biosystems). The 16S rRNA gene sequence was aligned with related sequences obtained from the GenBank database (National Center for Biotechnology Information, National Library of Medicine) using the BLAST search program. The 16S rRNA gene sequence of *Pseudomonas putida* TK1401 was deposited in GenBank (GenBank ID: AB362881).

### Thermographic assessments of bacterial colonies

To screen and isolate heat-producing bacteria, we measured the surface temperatures of bacterial colonies. Soil bacteria that had been stored at −80°C were inoculated in LB broth and incubated at 30°C for 12 hours. After this pre-incubation, 10 μl of the culture medium was inoculated onto LB agar plates that contained 1% (w/v) glucose. After incubation at 30°C for 2 days, the plates were placed on an aluminum block maintained at 30°C (Additional file 1: Figure S1). The plate covers were left open and the surface temperatures were measured using an infrared imager (Neo Thermo TVS-700, Nippon Avionics Co., Ltd), which had a temperature resolution of 0.08°C at 30°C Black Body (0.05°C or better with averaging).

To determine the temperature difference between a bacterial colony and the surrounding medium, we assessed the infrared images of the growth plates. Bacterial isolates were inoculated and incubated as above. The plate covers were then opened in the incubator and the surface temperature was measured using the infrared imager placed inside the incubator (Additional file 1: Figure S2). During these measurements, the plate was enclosed in a small chamber equipped with a window for thermographic measurements to avoid temperature fluctuations and airflow from the incubator. The temperature difference between a colony and the surrounding medium was determined from the average of the pixels in the infrared image. A typical infrared image is shown in Additional file 1: Figure S3.

We also examined the infrared images of colonies grown on a thermal gradient medium. The isolated bacteria stored at −80°C were inoculated in LB broth and incubated at 30°C for 12 hours. After this pre-incubation, 10 μl of the culture medium was inoculated on each 1 cm on LB agar plates (10 × 15 cm) that contained 1% (w/v) glucose. The medium plate was then placed upside down on a table, and a thermal gradient plate (thermal gradient gel electrophoresis system; TITEC Co., Japan) was placed on top of the LB agar plate. The temperature of the thermal gradient plate was controlled using two thermocirculator units. After incubation for 2 days under this thermal gradient, infrared images of the LB agar plate were assessed. The surface temperature of the medium was also measured using a thermocouple thermometer (Testo 950, Testo KK) connected to a super-quick action immersion/penetration probe (diameter = 1.5 mm), which had been calibrated using a highly accurate immersion/penetration probe. An infrared image was calibrated using the data from the thermocouple thermometer.

#### Growth rate determinations for strain TK1401 on LB agar

Strain TK1401 that had been stored at −80°C was inoculated in LB broth containing 1% (w/v) glucose and incubated at 30°C overnight. The turbidity of the culture medium was measured at 590 nm and diluted with LB broth containing 1% (w/v) glucose until its optical density at 590 nm was 0.01. Fifty microliters of this culture medium was inoculated onto LB agar plates that contained 1% (w/v) glucose, which were then incubated at 20.0, 22.5, 27.0, 30.0 32.5, and 35.0°C. After incubation, all bacterial cells that grew on the medium plates were harvested as follows. LB broth (1 ml) was poured and bacterial cells on the medium plates were suspended using a spreader. This suspension was collected from the medium plate. Another 1 ml of LB broth was poured on the medium plates and the suspension was collected from the medium plate. Both suspensions were collected and centrifuged at 2,000 *× g* for 10 min. The bacteria pellet was resuspended in 2 ml of LB broth. The turbidity of the suspension was measured at 590 nm, which was used as an estimate of the number of cells. Determination of the number of bacterial cells that grew on each medium plate was replicated thrice for each incubation time. The growth rate was calculated from the time-dependent changes in the number of bacterial cells that grew on each medium plate.

### Measurements of heat production and growth rates on LB agar using a microcalorimeter

Strain TK1401 that had been stored at −80°C was inoculated in LB broth containing 1% (w/v) glucose and incubated at 30°C overnight. The turbidity of the culture medium was measured at 590 nm and diluted with LB broth containing 1% (w/v) glucose until its optical density at 590 nm was 0.01. Ten microliters of this culture medium was inoculated on 2 ml of LB agar in a vial, and this vial was placed in a microcalorimeter (SuperCRC, OmiCal Technologies Inc.) to measure its heat output. The growth rate during the logarithmic growth phase was determined by the time-dependent change in heat output (Additional file 1: Figure S4) [17]. The heat output by a bacterial cell during the logarithmic growth phase was determined as follows. When the amount of heat output of the vial reached approximately 0.3–0.8 mW, the vial was removed from the microcalorimeter and all bacteria in the vial were suspended in LB broth. After pelleting and washing the bacterial cells with water, the amount of protein was determined using a DC protein assay kit (Bio-Rad Laboratories, Inc.). The heat output per mass of protein was then calculated.

## Results

**Figure 1:**
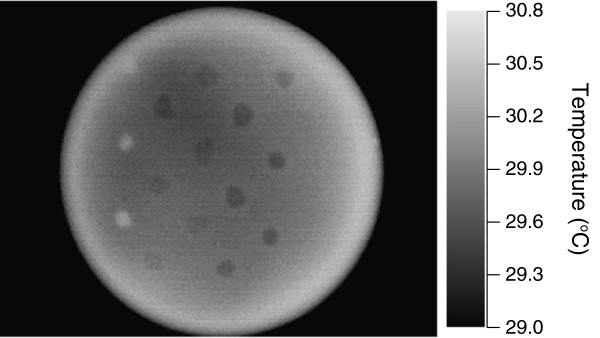
**Thermographs of bacterial colonies on growth plates after incubation for 2 days at 30°C.** Temperature on the thermographs is indicated by the color bar.

After culturing soil bacteria on LB agar plates containing 1% (w/v) glucose and incubating at 30°C for 2 days, the temperature of each colony was measured using an infrared imager. The thermographs of some colonies indicated that the colony temperatures were different from that of the surrounding medium (Figure 1). We measured the colony temperatures of 998 bacterial isolates from soils. The colony temperatures of 5 bacterial isolates were 0.1°C −0.2°C higher than that of the surrounding medium, suggesting that they increased the colony temperature above that of the surrounding medium. The colony temperatures of 421 bacterial isolates were lower than that of the surrounding medium, and the colony temperatures of the remaining isolates were similar to that of the medium. Strain TK1401 showed the highest colony temperature and was identified as *Pseudomonas putida* based on its 16S rRNA gene sequence.

**Figure 2:**
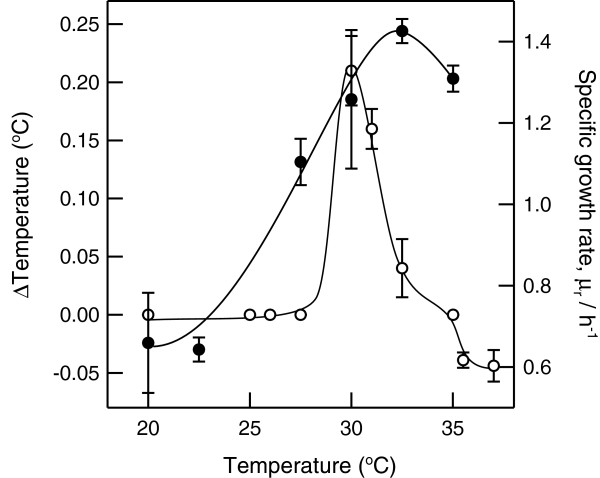
**Growth medium temperature dependence of the colony temperature and growth rate of *P. putida *TK1401.** Open circles: temperature difference between a bacterial colony and that of the growth medium; closed circles: specific growth rates. The temperature difference between the bacterial colony and that of the growth medium was determined from three replicates and is given as the mean ± standard deviation.

Heat production by bacteria is associated with their metabolic activity, which is affected by the incubation temperature. To investigate the effects of incubation temperature on colony temperature, the temperatures of *P. putida* TK1401 colonies were thermographically measured after incubation at varying temperatures. *P. putida* TK1401 could form colonies after incubation for 2 days at 20°C −37°C. We found that the colony temperature was 0.24°C higher than that of the surrounding medium when this bacterium was grown at approximately 30°C (Figure 2). As a control, we measured the colony temperature of bacteria exposed to chloroform vapor after incubation at 30°C for 2 days. The temperature of the colony comprising dead cells did not increase. Next, we investigated the relationship between the colony temperature and growth rate.

The growth rate of bacteria that grew on LB agar plates was determined based on the turbidity of cell suspensions harvested from the plate cultures. The sizes of bacterial cells were measured using Scanning electron microscopy (data not shown) because cell sizes affect the turbidity of a cell suspension. The cell size was approximately 0.4 × 1.2 μm and was not affected by the growth temperature. As shown in Figure 2, the optimal growth temperature for *P. putida* TK1401 was 32.5°C. Its colony temperature was similar to that of the surrounding medium, even at its optimal growth temperature. Although thermogenesis usually depends on bacterial growth, in the case of *P. putida* TK1401, an increase in colony temperature was only observed at a suboptimal growth temperature.

**Figure 3:**
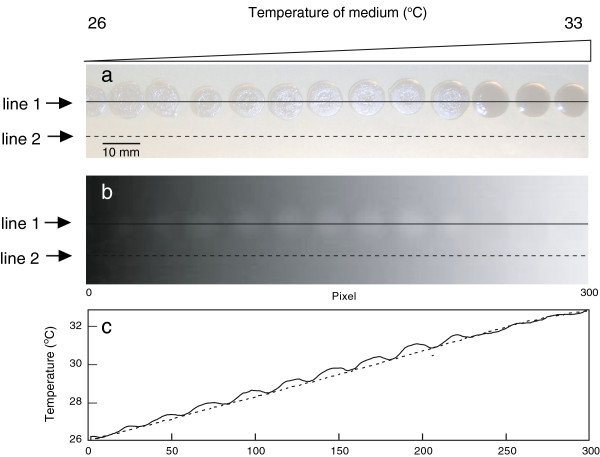
**A linear temperature gradient (26°C −33°C) was applied horizontally to a bacterial growth plate from left to right in the image. a**: Representative photograph of *P. putida* TK1401 grown on a thermal gradient plate. Bacterial cells were incubated for 2 days on the thermal gradient plate. Line 1 is drawn through the colonies and line 2 is only drawn through the medium. **b**: Representative thermographs of *P. putida* TK1401 grown on a thermal gradient plate. **c**: Temperature profiles of colonies and growth medium are shown by solid and dashed lines, respectively (lines 1 and 2, respectively, in Figure 3a and b).

Figure 3 shows thermograph and photograph of the bacterial colonies after 2 days of incubation at 26°C −33°C on thermal gradient plates. In this photograph, the temperature of the thermal gradient plate increased linearly from left to right. *P. putida* TK1401 formed colonies under these conditions (Figure 3a), and the colonies that grew at 30°C were more clearly visible in the thermograph compared with the colonies that grew at other temperatures (Figure 3b). Figure 3c shows the temperature profiles of the thermal gradient plate as determined by thermography. The colony temperature was higher than that of the growth medium at a growth temperature lower than 31.5°C, whereas it was similar to that of the growth medium at a growth temperature higher than 31.5°C. The colony temperature was approximately 0.4°C higher than that of the growth medium at a growth temperature of 30°C. Thus, *P. putida* TK1401 exhibited a unique thermal behavior when grown at approximately 30°C.

**Figure 4:**
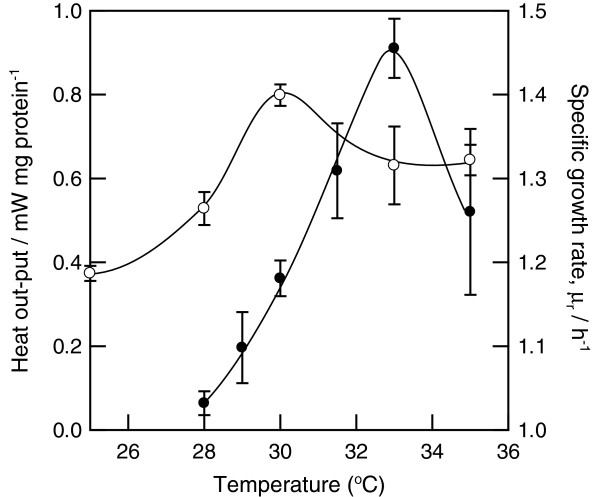
**Temperature dependence of the heat output and growth rate of *P. putida *TK1401.** Heat output and growth rate were determined using a microcalorimeter. Open circles: heat output from bacterial cells; closed circles: growth rates. Results are means ± standard deviations determined from three replicates.

This difference in colony temperature may have been related to bacterial thermogenesis, which was assessed by microcalorimetry. As shown in Figure 4, the highest heat output by the bacterial isolates was 0.8 mW/mg protein when cultures were incubated at 30°C. The temperature of this extraordinary, microcalorimetrically determined thermogenesis corresponded with the thermographically observed increase in bacterial colony temperature. These data suggested that the increase in colony temperature at 30°C was caused by increased thermogenesis by these bacterial cells. The growth rate of this strain on LB agar was also determined from the time-dependent changes in heat output. The optimal growth temperature of this bacterium in the microcalorimeter was 33°C. These data indicated that the extraordinary thermogenesis of *P. putida* TK1401 occurred at a suboptimal growth temperature.

**Figure 5:**
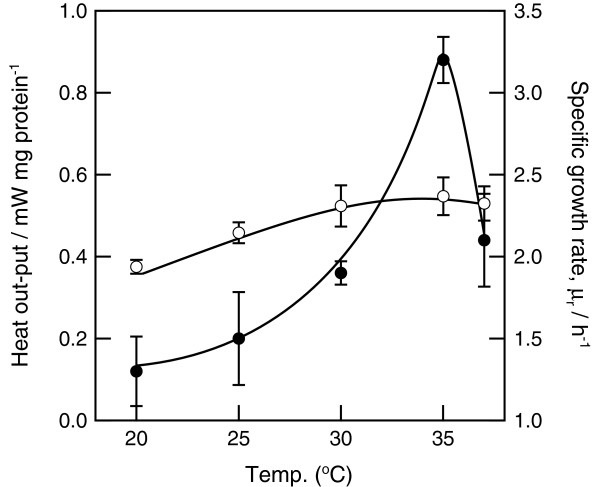
**Temperature dependence of the heat output and growth rate of *P. putida *TK2440.** Heat output and growth rate were determined using a microcalorimeter. Open circles: heat output from bacterial cells; closed circles: growth rates. Results are means ± standard deviations determined from three replicates.

To compare the heat production by *P. putida* TK1401 with the heat production by other bacteria, the heat output of *P. putida* KT2440 was measured. *P. putida* KT2440 is phylogenetically close to *P. putida* TK1401; however, it did not exhibit any increase in colony temperature. The heat production by this bacterium remained nearly constant when incubated at varying temperatures (Figure 5), which indicated that the heat output of *P. putida* KT2440 was independent of the growth temperature.

In order to produce excess heat, bacteria utilize more energy than that required for their growth. To investigate the effects of varying concentrations of an energy source on thermal behavior, the colony temperature and heat production of *P. putida* TK1401 were measured using varying concentrations of an energy source (Table 1). Colony temperature did not increase when this bacterium was grown on 0.25× and 0.5× LB media, but it did increase when this bacterium was cultured on 1×, 2×, and 5× LB agar plates. The highest colony temperature was observed when *P. putida* TK1401 was grown on 5× LB medium. These data indicated that the colony temperature of *P. putida* TK1401 increased under energy-rich conditions.

**Table 1:** **Effects of energy source on *P. putida *TK1401 colony temperature**

**Medium**	**ΔTemperature**^**a**^	**Heat output**^**b**^	**Specific growth rate**^**b**^
	**(°C)**	**(mW mg protein**^**−1**^**)**	**(h**^**−1**^**)**
0.25× LB medium	0.00 ± 0.00	0.62 ± 0.00	1.3 ± 0.1
0.5× LB medium	0.00 ± 0.00	0.70 ± 0.10	1.4 ± 0.1
1× LB medium	0.24 ± 0.17	0.82 ± 0.03	1.2 ± 0.0
2× LB medium	0.22 ± 0.15	0.88 ± 0.03	1.4 ± 0.1
5× LB medium	0.37 ± 0.20	0.93 ± 0.05	1.3 ± 0.1

^a^Temperature difference between a colony and growth medium.

^b^Heat output and specific growth rate were determined using a microcalorimeter. Results are means ± standard deviations determined from three replicates.

The heat output from this bacterium also increased as the concentration of the energy source in the medium increased. In contrast, the growth rate of this bacterium was constant under these conditions. Thus, the 0.25× and 0.5× LB agar plates also contained sufficient energy for *P. putida* TK1401 growth at its maximum growth rate. These results indicated that this bacterium produced excess heat when the energy source was in excess. When this bacterium was incubated at varying temperatures on 0.25× LB medium, no increase in colony temperature was observed and the heat output from this bacterium was not altered by the growth temperature (Additional file 1: Table S1). When this bacterium was grown on 0.25× LB medium at varying temperatures, its heat output was the same as those when grown on LB medium that contained 1% glucose, except at 30°C.

These results suggested that the heat output from the growth-dependent reaction was approximately 0.6 mW and that the heat output from the growth-independent reaction was approximately 0.3 mW when this bacterium was grown at 30°C on 5× LB medium.

## Discussion

Some insects and plants increase their body temperatures using the heat generated from metabolic reactions [18-21]. However, the cellular temperatures of microorganisms have not been measured and the effects of metabolic reactions on their cellular temperatures have not been previously investigated. In this study, we measured the temperatures of bacterial colonies using thermography. This revealed that the temperatures of some bacterial colonies differed from that of their surroundings. In particular, the isolated bacterium *P. putida* TK1401 could maintain a colony temperature that was higher than that of the surrounding medium. These results indicate that some bacteria are capable of maintaining a cellular temperature that is different from the ambient temperature.

We isolated the bacterium *P. putida* TK1401 that could maintain a temperature higher than that of the surrounding medium when it was incubated at 30°C and generated a heat output of 0.8 mW/mg protein. This heat output was high compared with the heat output of *P. putida* TK1401 grown at other temperatures and that of *P. putida* KT2440. These results suggest that the heat production by bacteria affects the colony temperature and that some bacteria can maintain a cellular temperature different from the ambient temperature.

The amount of heat produced by *P. putida* TK1401 changed depending on the growth temperature and the concentration of a nutrient (Figure 4 and Table 1). The greatest heat production was observed when this bacterium was incubated on 5× LB agar medium at 30°C. Under these conditions, the amount of heat produced by *P. putida* TK1401 was greater than the amount of heat produced by metabolic reactions for growth. Therefore, this bacterium consumed energy to produce heat without producing additional biomass at 30°C. These results suggest that this increase in thermogenesis was caused by a growth-independent reaction.

The energy-spilling reactions of some bacteria occur under conditions of limited nitrogen and an excess energy source [9-12]. *P. putida* TK1401 produced excess heat when it was incubated at a temperature lower than its optimal growth temperature. When this bacterium was incubated at 30°C, the heat production increased as the concentration of nutrient increased. Under these conditions, there were sufficient amounts of nutrients for its growth, although this temperature limited the growth of this bacterium. Thus, the energy-spilling reaction of *P. putida* TK1401 may be induced under temperature-limiting conditions.

An increase in colony temperature was only observed between 27°C and 31°C, which are suboptimal growth temperatures for *P. putida* TK1401. At temperatures less than 27°C, the colony temperatures and heat production of this bacterium did not increase. The enzymes that are related to heat production may have been induced at incubation temperatures between 27°C and 31°C or the specific activities of these enzymes may have been too low to affect the colony temperature and the amount of heat production at temperatures less than 27°C.

Energy-spilling reactions are mediated by futile cycles. Some mechanisms involving futile cycles have been proposed for bacteria, including (1) futile cycles of enzymes involved in phosphorylation and dephosphorylation [13] and (2) futile cycles of membrane transfer, such as potassium ions, ammonium ions, and protons [22-24]. The mechanism of a futile cycle that mediates the heat production by *P. putida* TK1401 is unknown. The previously reported energy-spilling reactions of bacteria were activated under nutrient-limited and excess energy source conditions. The heat production by *P. putida* TK1401 increased under nutrient-rich conditions. Thus, the futile cycle of *P. putida* TK1401 could be related to nitrogen availability such as through the urea cycle.

## Conclusion

We measured the colony temperatures of soil bacteria using thermography and found that the temperatures of some colonies were higher or lower than that of the surrounding medium. The bacterial isolate with the highest colony temperature, KT1401, was identified as *Pseudomonas putida*. The colony temperature of *P. putida* KT1401 increased when isolates of this bacterium were grown at a suboptimal growth temperature. Heat production by this bacterium increased without the production of additional biomass at a suboptimal growth temperature. Therefore, *P. putida* KT1401 may convert energy into heat by an energy-spilling reaction when the incubation temperature limits its growth.

## Authors’ contributions

Conception and design: KT, IO. Methodology development: KT, FH, TK. Data acquisition: FH, TK, NI. Data analysis and interpretation: KT, FH, Manuscript writing, review, and/or revision: KT, TK, IO. All authors read and approved the final manuscript.

## Supplementary Material

Additional file 1: Table S1 Colony temperature and heat output of *P. putida* TK1401 grown on low energy source medium. **Figure S1.** The equipment for the measurement of the infrared image of the bacterial colonies. **Figure S2.** The equipment for the measurement of the temperature differences between the bacterial colony and the surrounding medium. **Figure S3.** Thermograph of bacterial colonies of *P. putida* KT1401 on medium plate after incubation for 2 days at 30°C. The temperature on the thermographs is indicated by the color bar. **Figure S4.** Typical data relating to time-dependent changes in heat output of *P. putida* TK1401. The bacterium grew at 30°C on LB agar medium in a vial. Heat output was measured using a microcalorimeter. The insert is a semi-logarithmic plot of the heat output. Click here for file
