# Supplementary material for: Measurement of soil bacterial colony temperatures and isolation of a high heat-producing bacterium
Source: BMC Microbiol. 2013 Mar 11;13:56. doi: 10.1186/1471-2180-13-56 (PMC3608088; doi:10.1186/1471-2180-13-56)
Supplement: Additional file 1: Table S1 — Colony temperature and heat output of P. putida TK1401 grown on low energy source medium. Figure S1. The equipment for the measurement of the infrared image of the bacterial colonies. Figure S2. The equipment for the measurement of the temperature differences between the bacterial colony and the surrounding medium. Figure S3. Thermograph of bacterial colonies of P. putida KT1401 on medium plate after incubation for 2 days at 30°C. The temperature on the thermographs is indicated by the color bar. Figure S4. Typical data relating to time-dependent changes in heat output of P. putida TK1401. The bacterium grew at 30°C on LB agar medium in a vial. Heat output was measured using a microcalorimeter. The insert is a semi-logarithmic plot of the heat output. [file 1471-2180-13-56-S1.doc]

**Table**

Additional file 1: Table S1 Colony temperature and heat output of *P. putida* TK1401 grown on low energy source medium

| Growth temperature  /°C | ΔTemperature^a^  / °C | Heat output^b^  / mW mg protein^-1^ | Specific growth rate^b^  / h^-1^ |
| --- | --- | --- | --- |
| 26 | 0.00 | 0.57±0.00 | 1.0±0.1 |
| 28 | 0.00 | 0.62±0.01 | 1.2±0.2 |
| 30 | 0.00 | 0.62±0.00 | 1.3±0.1 |
| 32 | 0.00 | 0.67±0.03 | 1.4±0.1 |
| 34 | 0.00 | 0.67±0.05 | 1.2±0.1 |

The bacterium was inoculated on 0.25 x LB medium.

^a^Temperature difference between a colony and growth medium.

^b^Heat output and specific growth rate were determined using a microcalorimeter. Results are means ± standard deviations determined from three replicates.

**Figure**


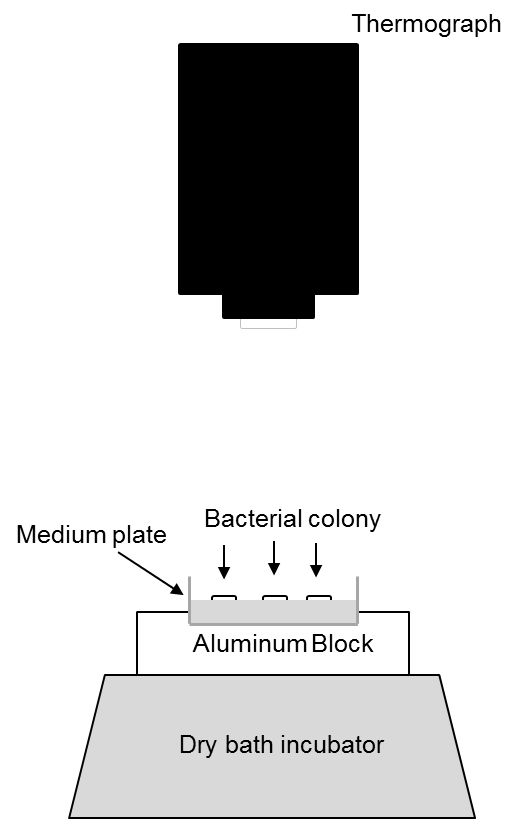


Additional file 1: Figure S1 The equipment for the measurement of the infrared image of the bacterial colonies.


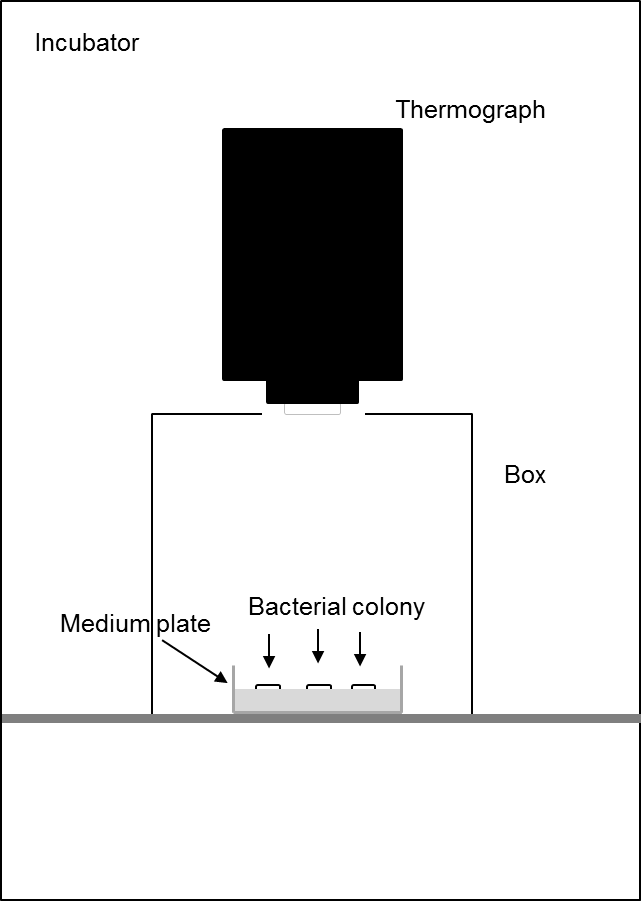


Additional file 1: Figure S2 The equipment for the measurement of the temperature differences between the bacterial colony and the surrounding medium.


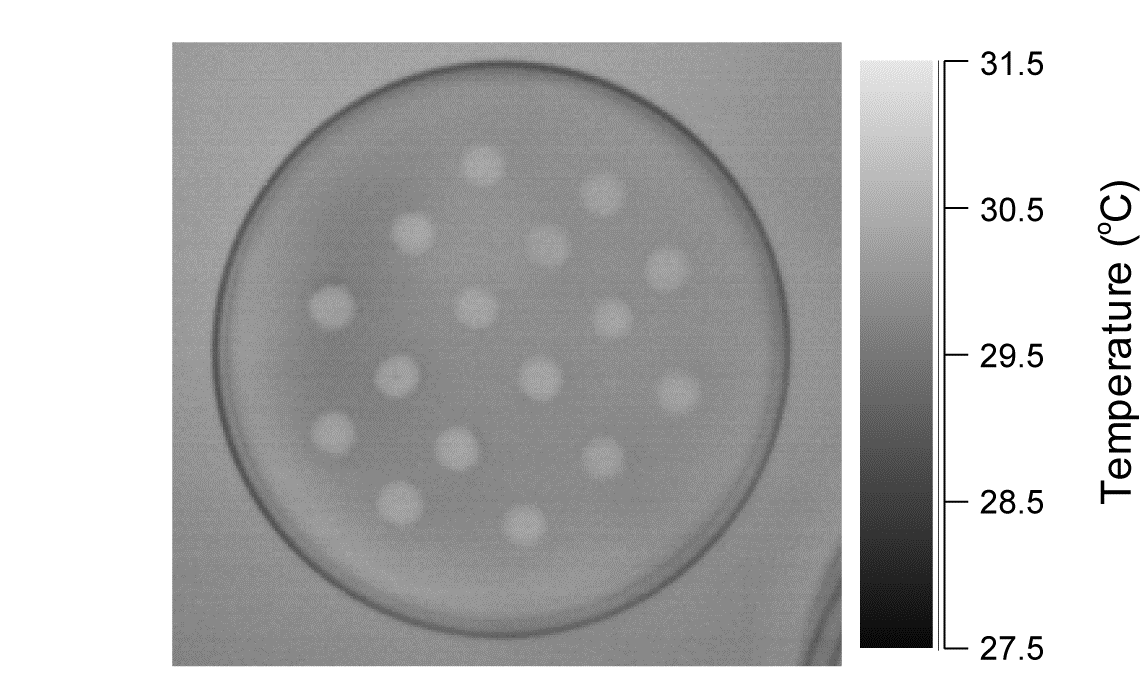


Additional file 1: Figure S3 Thermograph of bacterial colonies of *P. putida* KT1401 on medium plate after incubation for 2 days at 30°C. The temperature on the thermographs is indicated by the color bar.

Additional file 1: Figure S4 Typical data relating to time-dependent changes in heat output of *P. putida* TK1401. The bacterium grew at 30°C on LB agar medium in a vial. Heat output was measured using a microcalorimeter. The insert is a semi-logarithmic plot of the heat output.
